# Instruments Used to Assess Health-Related Quality of Life in Valvular Heart Disease: A Narrative Review

**DOI:** 10.7759/cureus.94376

**Published:** 2025-10-12

**Authors:** Andra D Marinescu, Victor S Costache

**Affiliations:** 1 Faculty of Medicine, Titu Maiorescu University, Bucharest, ROU

**Keywords:** eq-5d, health-related quality of life, hrqol instruments, kccq, mlhfq, patient-reported outcomes, quality of life measurement, sf-36, valvular heart disease, whoqol-bref

## Abstract

Health-related quality of life (HRQoL) has become a pivotal outcome in valvular heart disease (VHD), complementing mortality and readmission. This narrative (non-systematic) review synthesizes validated HRQoL instruments used in VHD, spanning generic tools - the Short Form-36 and derivatives (SF-12, SF-6D), EuroQol 5-Dimensions (EQ-5D), and World Health Organization Quality of Life - Brief (WHOQOL-BREF) - and disease-specific measures such as the Minnesota Living with Heart Failure Questionnaire (MLHFQ) and Kansas City Cardiomyopathy Questionnaire (KCCQ). Instruments were included if (i) applied to adult VHD populations or valve interventions or (ii) demonstrated use in VHD cohorts; English-language sources (primarily 2015-2025) were prioritized, retaining seminal pre-2015 validation papers. Pediatric-only cohorts, non-validated tools, and case reports were excluded.

MLHFQ and KCCQ were included on an evidence basis: both have been validated in valvular populations and used as key endpoints in pivotal interventional studies, demonstrating high reliability and sensitivity to change after surgical aortic valve replacement (SAVR), transcatheter aortic valve replacement (TAVI), and transcateter edge-to-edge repair (TEER). Their established minimal clinically important differences and the availability of a short form (KCCQ-12) further support feasibility and interpretability in practice. The review is descriptive and therefore psychometric properties (validity, reliability, responsiveness, minimal clinically important differences), covered domains, feasibility, and cultural/linguistic adaptation are summarized from existing studies. Emphasis is placed on domains most germane to VHD recovery - physical function, symptom burden, emotional well-being, and social/role participation - given their sensitivity to peri-interventional change.

Recommendations for clinical integration (e.g., pairing a generic utility measure such as EQ-5D with a disease-specific tool such as KCCQ; standardized assessment at baseline, 30 days, six and 12 months) are evidence-informed (randomized trials, registries, guidelines). Key gaps include limited valve-specific content (e.g., prosthesis noise, anticoagulation burden), heterogeneity in instruments and time points, incomplete cultural validation, and scarce HRQoL data for emerging therapies (e.g. transcatheter mitral valve replacement, contemporary tricuspid interventions). As a narrative (non-systematic) purely descriptive synthesis, no formal risk-of-bias assessment or meta-analysis was conducted. The English-language restriction and heterogeneity across instruments and follow-up time points limit quantitative comparability and preclude definitive head-to-head conclusions. Future work should develop core outcome sets and VHD-specific modules, strengthen cross-cultural validation, and leverage digital patient-reported outcome platforms to improve comparability and clinical uptake.

## Introduction and background

Valvular heart disease (VHD) represents an important and growing health burden worldwide, particularly among older adults and in regions where rheumatic heart disease remains prevalent [1]. The prevalence of moderate-to-severe VHD increases sharply after the age of 65, reaching approximately 2%-4% in individuals aged 65-75 years. Degenerative conditions, such as calcific aortic stenosis and mitral regurgitation, compromise cardiac function and are the major contributors to morbidity and mortality.

In this clinical context, the concept of health-related quality of life (HRQoL) has gained significant relevance as a key patient-centered outcome, especially in chronic cardiovascular illnesses and in the evaluation of post-surgical recovery [2]. HRQoL is a multidimensional construct that reflects the individual’s perception of their physical, emotional, and social well-being, extending beyond traditional clinical endpoints. As interventions for VHD - whether surgical or transcatheter - aim not only to prolong survival but also to improve daily functioning and overall well-being, the assessment of HRQoL offers an essential insight into the perceived effectiveness of treatment [3].

Several validated questionnaires are available for the measurement of HRQoL, which can be broadly classified into generic and disease-specific categories. Multiple instruments coexist because they balance different priorities - comparability across conditions (generic), sensitivity to valve-related change (disease-specific), and feasibility or utility generation for health-economic analyses-so no single tool fits all VHD contexts. The Short Form-36 Health Survey (SF-36) [4] is the most widely used generic instrument and has been validated across a variety of cardiac populations, including those with heart failure. It evaluates multiple domains such as physical functioning and mental health. Another widely applied generic tool is the Euro Quality of Life 5 Dimensions (EuroQoL-5D), valued for its brevity and its applicability in health economic evaluations [5]. Although originally developed for heart failure, the Minnesota Living with Heart Failure Questionnaire (MLHFQ) and the Kansas City Cardiomyopathy Questionnaire (KCCQ) have been repeatedly applied - and, in several cohorts, formally evaluated - in valvular settings, where they demonstrate high reliability and sensitivity to peri-interventional change; established minimal clinically important differences further support interpretability in VHD populations [6].

Among the disease-specific instruments, the MLHFQ has demonstrated particular sensitivity and validity in patients undergoing heart valve surgery, often outperforming generic instruments in detecting subtle post-intervention changes [6]. Nevertheless, the sensitivity, practicality, and suitability of these tools vary across VHD populations. For example, patient responses may differ depending on whether the intervention is surgical or transcatheter, and while instruments such as the SF-36 offer comprehensive assessment, they may be too lengthy for routine clinical application; in contrast, shorter tools may sacrifice disease-specific sensitivity [7]. In addition, current instruments incompletely capture valve-specific issues (e.g., prosthesis noise, anticoagulation burden, device-related anxiety), underscoring the need for VHD-tailored modules alongside generic and disease-specific measures [2,3].

This review synthesizes the most commonly used generic and disease-specific HRQoL instruments in VHD, evaluating their psychometric properties, clinical utility, and relevance in both surgical and transcatheter settings. The aim is to provide clinicians and researchers with practical guidance for selecting appropriate tools for patient-centered outcome measurement in valvular heart interventions. All instruments are described based on previously published sources; no questionnaires were administered or reproduced in full within this article. Because value sets and costing inputs for EuroQol 5-Dimensions (EQ-5D) are country-specific, cost-utility results are not universally transferable; interpretation should consider the healthcare system context and local tariffs [5]. Throughout, HRQoL is considered both a prognostic indicator associated with clinical outcomes and a decision-informing patient-reported outcome that complements, but does, under no circumstance, replace guideline-based clinical criteria in shared decision-making.

Overview of HRQoL and its importance in VHD

HRQoL is a multidimensional concept that encompasses an individual’s subjective perception of how their physical, psychological, and social health status affects overall well-being and daily functioning. Unlike general quality of life (QoL), which includes broader aspects such as financial security, spirituality, or environmental conditions, HRQoL focuses specifically on the elements directly or indirectly influenced by health and disease [8].

The concept emerged during the 1970s and 1980s when clinicians and researchers began to recognize that conventional clinical endpoints - such as mortality rates, hospitalization, or laboratory values - were insufficient to fully describe the patient’s experience of illness and treatment [9]. In 1980, Patrick and Erickson offered one of the earliest structured definitions, describing HRQoL as “the value assigned to duration of life as modified by the impairments, functional states, perceptions, and social opportunities that are influenced by disease, injury, treatment, or policy" [9]. Since then, HRQoL has become integral to patient-centered care, forming the basis for patient-reported outcome measures (PROMs) used in clinical trials, public health programs, and healthcare policy [10,11]. Cross-cultural and regional differences in health perception are particularly relevant where rheumatic VHD predominates; instruments require culturally adapted, language-validated versions and attention to potential differential item functioning to ensure comparability across settings [9].

This framework is rooted in the biopsychosocial model, which recognizes that health outcomes are influenced not only by biological factors but also by psychological and social determinants [3]. The World Health Organization (WHO) has been a strong advocate for integrating QoL into health assessment, defining it as “an individual’s perception of their position in life in the context of the culture and value systems in which they live” [12].

In the context of measurement, HRQoL is commonly conceptualized through several key domains, including physical functioning (mobility, energy levels, pain), psychological well-being (mood, anxiety, cognitive function), social relationships (social support, role limitations), role functioning (ability to fulfill work or home responsibilities), and perceived general health [2,3]. These domains guide the development of measurement instruments, such as the SF-36 or EQ-5D, and inform the interpretation of their results in clinical research. Importantly, HRQoL assessment captures not only the presence or absence of symptoms but also the patient’s personal interpretation and adaptation to their condition, which can differ significantly between individuals with similar medical profiles [7]. Domain emphasis may also differ by treatment pathway: after surgical valve replacement, measures capturing pain reduction and recovery of physical functioning (role-physical domain) are informative, whereas after transcatheter procedures, early changes in mobility, self-care, and symptom burden are typically most responsive; shorter tools (e.g., EQ-5D, KCCQ-12) can improve feasibility in older or frail cohorts [8].

Modern approaches to HRQoL recognize its dynamic nature: as patients progress through different stages of disease, treatment, and recovery, the relative weight of each domain may shift. Consequently, longitudinal assessments are preferred over single-point evaluations [8]. In elderly or frail patients, longitudinal HRQoL assessment is vulnerable to missing data, recall bias, sensory/cognitive limitations, and mode-of-administration effects; where capacity is limited, carefully documented proxy assessments may be necessary, acknowledging only moderate agreement with self-report and the need for cautious interpretation [13]. Furthermore, HRQoL has increasingly been shown to predict hard clinical outcomes. Studies have demonstrated that lower baseline HRQoL scores are associated with increased risk of hospitalization, poorer treatment adherence, and higher mortality rates in chronic conditions, including cardiovascular diseases [7]. In valve populations specifically, baseline and early post-procedural HRQoL track with subsequent functional recovery and survival after both surgical and transcatheter interventions [3].

In VHD specifically, the role of HRQoL is particularly significant. Many interventions, such as surgical or catheter-based valve repair or replacement, are primarily aimed at relieving symptoms like dyspnea and fatigue rather than reversing the structural damage of the valve. In elderly or frail populations, procedures such as transcatheter aortic valve implantation (TAVI) may offer modest survival benefits but deliver substantial gains in perceived quality of life, independence, and emotional well-being [13]. Furthermore, many patients adapt over time to chronic symptoms, leading to underreporting unless HRQoL is assessed systematically [7].

HRQoL data also play a central role in health economic evaluations. Cost-effectiveness studies comparing TAVI with surgical aortic valve replacement, or mechanical with biological prostheses, often rely on quality-adjusted life years (QALYs), which are derived directly from validated HRQoL instruments such as the EQ-5D [14]. Therefore, HRQoL outcomes influence not only patient-level decisions but also broader policy and resource allocation strategies [9,15]. Given variability in valuation sets and costing inputs, such cost-utility analyses are inherently context-dependent and should be interpreted within the relevant health-system framework [5,14].

Finally, incorporating HRQoL assessments into routine follow-up enables clinicians to monitor recovery holistically, tailor rehabilitation programs, and identify unaddressed needs such as anxiety, depression, or social isolation-factors that may be overlooked in conventional follow-up but are critical for long-term success [16].

## Review

Methods (narrative review approach)

A targeted, narrative review of the literature was conducted in early-mid 2025. Medline (PubMed) and Google Scholar were searched using combinations of keywords and medical subject headings (MeSH)/Emtree terms covering (i) valvular disease and related interventions and (ii) health-related quality of life and patient-reported outcomes, together with names of specific instruments. A representative PubMed query was: (“valvular heart disease” OR “aortic stenosis” OR “mitral regurgitation” OR “tricuspid regurgitation” OR SAVR OR TAVI OR “transcatheter aortic valve implantation” OR TEER OR MitraClip OR TMVR) AND (“quality of life” OR “health-related quality of life” OR HRQoL OR “patient-reported outcome” OR PROMs) AND (SF-36 OR “Short Form 36” OR “SF-12” OR “SF-6D” OR EQ-5D OR “EuroQol” OR WHOQOL-BREF OR MLHFQ OR “Minnesota Living with Heart Failure Questionnaire” OR KCCQ OR “Kansas City Cardiomyopathy Questionnaire” OR HeartQoL) (SAVR: surgical aortic valve replacement; TAVI: transcatheter aortic valve replacement; TEER: transcatether edge-to-edge repair; TMVR: transcatether mitral valve replacement).

English-language studies and reviews reporting HRQoL/PROMs in adult valvular populations or in valve interventions were included, in addition to validation studies of commonly used HRQoL instruments. Pediatric-only cohorts, non-cardiac populations, case reports without HRQoL data, and non-validated or unpublished tools were excluded.

Titles and abstracts were screened by a single reviewer, with full-text assessment to confirm eligibility. Consistent with the narrative scope, we did not perform meta-analysis or a formal risk-of-bias appraisal; instead, when summarizing effects, we preferentially weighted higher-quality designs (randomized trials, large registries, and systematic reviews). We performed a narrative synthesis of relevant studies rather than a tabulated data extraction. Our emphasis was on populations and contexts, HRQoL instruments (domains and scoring), psychometric properties (validity, reliability, responsiveness), and the principal HRQoL results. Searches were last refreshed in September 2025 to capture recent developments.

Assessment instruments

HRQoL in patients with VHD can be measured using either generic or disease-specific instruments, each with distinct advantages and limitations. Generic instruments are standardized tools that assess a broad spectrum of physical, emotional, and social domains without focusing on a particular condition. Their versatility allows them to be applied across multiple patient populations, making it possible to compare outcomes between different diseases, interventions, or even with the general population [7,8]. In VHD, generic measures facilitate benchmarking and health-economic analyses; however, because they may miss valve-specific change, they are best used in combination with a disease-specific tool to balance feasibility, comparability, and sensitivity to clinical change [3,8,17].

Generic Instruments

In the context of VHD, generic instruments are valuable because they capture the global impact of the disease and its treatment, going beyond purely physiological measures [8]. While disease-specific questionnaires provide detailed insights into cardiac symptoms and functional limitations, generic tools also assess areas such as mental health, pain, vitality, and social functioning, thereby offering a more holistic view of patient well-being before and after interventions like surgical valve replacement or transcatheter aortic valve implantation (TAVI) [17]. Direct head-to-head comparisons between generic tools in VHD-only cohorts are limited; broadly, SF-36 provides richer domain profiling and tends to be more sensitive to postoperative functional recovery, whereas EQ-5D enables utility estimation for cost-effectiveness but can show ceiling effects after successful interventions [3,4,10,12].

Several generic instruments have been widely used in studies involving VHD patients, including the Short Form-36 (SF-36), Short Form-12 (SF-12), Short Form-6D (SF-6D), the EuroQol EQ-5D, and the World Health Organization Quality of Life - Brief (WHOQOL-BREF) [3,8]. These tools differ in length, domains covered, and scoring methods, and the choice of instrument depends on the clinical setting, research objectives, and the administrative burden it may impose. Cultural and language adaptation are critical in multinational or low-resource settings; validated translations, prespecified country tariffs (for EQ-5D), and attention to mode of administration are required to ensure measurement equivalence in older adults with varying health literacy [5,18].

SF-36 remains the most extensively used generic instrument in both general and clinical populations. Developed in the late 1980s as part of the Medical Outcomes Study, it evaluates eight domains - (i) physical functioning, (ii) role limitations due to physical health, (iii) bodily pain, (iv) general health, (v) vitality, (vi) social functioning, (vii) role limitations due to emotional problems, and (viii) mental health. These domains can be combined into two summary scores: the Physical Component Summary (PCS) and the Mental Component Summary (MCS), both standardized on a scale from 0 to 100, with higher scores indicating better perceived health. The SF-36 has proven to be highly applicable in VHD, capturing postoperative recovery patterns, changes in functional status, and the psychosocial impact of chronic valve disease [4,10]. Minimal clinically important differences (MCIDs) specific to VHD are not firmly established; many studies borrow cardiac/general thresholds and anchor interpretation to functional class or clinical events. Its main limitations are the length of the questionnaire and the potential burden on elderly or frail patients, especially those with cognitive decline [12].

SF-12 was developed as a concise alternative, containing only 12 items while still providing PCS and MCS scores comparable to those of the SF-36 [10]. Its brevity makes it well-suited for large-scale surveys and elderly populations. However, it does not produce scores for each of the eight domains individually, focusing solely on the physical and mental composite scores, and VHD-specific MCIDs have not been defined.

SF-6D is a preference-based measure derived from the SF-36, allowing for the calculation of health utility scores that are particularly useful in cost-utility analyses. It defines six dimensions - (i) physical functioning, (ii) role limitations, (iii) social functioning, (iv) pain, (v) mental health, and (vi) vitality - each with multiple levels, leading to over 18,000 possible health states, which are assigned utility values between 0.29 and 1.00. While valuable for economic evaluation, the SF-6D may be less sensitive to small clinical changes and may overemphasize physical functioning at the expense of psychosocial aspects [12]. In elderly VHD populations with multimorbidity, utilities can be driven by non-valvular conditions, narrowing detectable treatment effects; the relatively compressed utility range can also attenuate apparent gains after intervention.

The EuroQol EQ-5D is another widely used generic tool, notable for its simplicity and inclusion of a visual analogue scale (VAS) for self-rated health. It assesses mobility, self-care, usual activities, pain or discomfort, and anxiety or depression, with either three levels (EQ-5D-3L) or five levels (EQ-5D-5L). Its brevity and ease of administration make it particularly attractive for elderly populations, though ceiling effects can limit its sensitivity in patients with mild disease or soon after effective procedures [4]. Responsiveness in VHD is generally acceptable for early post-intervention gains, but VHD-specific MCIDs are heterogeneous and often inferred from broader cardiovascular cohorts. Interpretation in multinational studies should prespecify the country-specific value set and use validated translations to maintain cross-cultural comparability.

Finally, the WHOQOL-BREF, developed by the World Health Organization, offers a culturally adaptable approach to quality-of-life assessment. It covers physical health, psychological health, social relationships, and environmental factors, with each domain scored using a five-point Likert scale. Although less frequently applied in VHD compared to SF-36 or EQ-5D, this pattern reflects both clinical practicality (lack of preference-based utilities and limited peri-interventional responsiveness data in VHD) and validation scope (fewer VHD-specific studies). It remains useful when broader psychosocial/environmental contexts are central - particularly in multicultural or low-resource settings - provided language-specific validation is ensured [5,18].

Practical considerations for generic tools in VHD. Ceiling/floor effects in older or frail patients can blunt the observed change and should be mitigated by pairing a generic tool with a sensitive disease-specific instrument; overlapping domains across instruments mean study designs should avoid redundant combinations that add respondent burden without analytic value. In routine VHD applications, generic tools alone are rarely sufficient; a pragmatic pairing (e.g., EQ-5D for utilities plus a disease-specific measure for sensitivity) improves interpretability and supports both clinical and economic decision-making [3,8,17].

Disease-specific Instruments

While generic HRQoL instruments are valuable for comparing outcomes across different diseases and populations, they may lack the sensitivity to detect subtle but clinically important changes in patients with valvular heart disease. In these situations, disease-specific questionnaires offer a more targeted evaluation of symptoms, functional limitations, and emotional consequences directly related to cardiovascular pathology [9,18]. In this section, instruments originally developed for heart failure that are frequently used in VHD are described, and their validation status within valve populations, responsiveness, and practical considerations are clarified.

These specialized tools are often developed with direct input from patient populations and are designed to reflect the natural history, treatment effects, and prognostic implications of the disease more precisely than generic instruments. Their items typically address issues such as dyspnea, fatigue, physical limitations, emotional distress, social interaction, and the perceived burden of treatment domains that are highly relevant for individuals with symptomatic VHD, particularly those undergoing valve repair or replacement procedures [10]. However, because content was largely derived from heart failure cohorts, certain valve-specific concerns (e.g., prosthesis noise, anticoagulation burden, peri-operative issues) may be underrepresented, which can limit content validity for “pure” VHD without concomitant heart failure.

In patients treated with surgical valve replacement (SAVR) or transcatheter aortic valve implantation (TAVI), disease-specific questionnaires often demonstrate superior responsiveness to clinical changes compared to generic tools, providing a more accurate picture of recovery trajectories [14,16]. Among the most commonly used disease-specific measures in cardiovascular medicine are the Minnesota Living with Heart Failure Questionnaire (MLHFQ) and the Kansas City Cardiomyopathy Questionnaire (KCCQ). Although these were initially validated in heart failure populations, they are increasingly applied in valvular cohorts, especially when left ventricular dysfunction or overlapping symptoms are present [19]. Evidence specific to VHD includes formal psychometric evaluations in valve surgery and aortic stenosis cohorts for both MLHFQ and KCCQ, with high internal consistency and change sensitivity reported; nonetheless, head-to-head comparisons between these instruments and valve-specific tools in VHD-only samples remain limited, and most data come from single-condition or mixed cardiac studies [20-25].

The MLHFQ contains 21 items designed to assess how heart failure and its treatment have affected various aspects of the patient’s life over the preceding four weeks. Each item is scored on a six-point scale from 0 (“no impact”) to 5 (“high impact”), yielding a total score between 0 and 105, where lower scores indicate better HRQoL [20]. The questionnaire covers physical limitations such as shortness of breath and fatigue, emotional states including anxiety and depression, and social or functional restrictions such as inability to work or perform household activities. Psychometric testing has shown the MLHFQ to have high internal consistency (Cronbach’s α >0.90) and excellent responsiveness to clinical change, particularly after therapeutic interventions like valve surgery. Its minimal clinically important difference (MCID) is estimated at about five points [21]. It should be noted that this five-point MCID derives largely from heart failure literature; VHD-specific anchor-based thresholds have not been firmly established, so clinical interpretation in valve-only cohorts should be made cautiously and, where possible, supplemented by distribution-based estimates within the study sample. From a feasibility standpoint in older or frail valve patients, completion burden can be non-trivial; assisted administration or the use of a briefer companion tool may improve data completeness.

The KCCQ is another widely used disease-specific instrument. The full version contains 23 items organized into domains such as physical limitation, symptom frequency, symptom burden, self-efficacy, quality of life, and social limitation. Each domain and the overall summary score range from 0 to 100, with higher scores representing better health status [20]. The KCCQ has demonstrated high reliability (Cronbach’s α >0.80) and strong sensitivity to clinical change, making it one of the preferred tools for evaluating patient outcomes after interventions such as TAVI or mitral valve repair [23,24]. In the Cardiovascular Outcomes Assessment of the MitraClip Percutaneous Therapy for Heart Failure Patients with Functional Mitral Regurgitation (COAPT) trial, for instance, patients with secondary mitral regurgitation treated with transcatheter edge-to-edge repair experienced clinically meaningful improvements in KCCQ scores - often greater than 10 points - correlating with survival and hospitalization benefits [25]. A shortened version, the KCCQ-12, is also available; it maintains strong correlation with the original tool and is more practical for routine clinical use [23]. KCCQ has been applied and psychometrically supported in aortic stenosis (including surgical) and TAVI cohorts, but, similar to MLHFQ, VHD-exclusive validation datasets remain fewer than heart failure datasets. Commonly cited MCIDs (e.g., five to 10 points) originate from heart failure and device trials; VHD-specific MCIDs are not yet standardized. Given the administration time and cognitive load in elderly populations, KCCQ-12 is generally preferable for routine practice, reserving the full KCCQ for research requiring granular domain profiling.

Although both the MLHFQ and KCCQ are highly useful in valvular populations, they were not originally developed to address valve-specific concerns such as prosthesis-related anxiety, the burdens of anticoagulation therapy, or the impact of valve sound. Mechanical-valve patients often experience subjective discomfort from valve noise, which correlates with lower SF-36 scores and heightened anxiety [16]. To address this gap, researchers have developed proprietary instruments such as the Heart Valve Disease Impact on Daily Life (IDCV) tool, tailored to the valvular disease population's needs [26]. Hybrid approaches, where generic HRQoL instruments such as the SF-36 are supplemented with valve-specific items addressing issues like anticoagulation burden or prosthetic valve noise, have been applied in patients, for example, after mechanical mitral valve replacement [27]. These methods provide a more comprehensive assessment of patient experience, but they are still in preliminary use and require further standardization and psychometric validation [28,29]. Current reports suggest the IDCV has undergone initial development with face/content validity and internal consistency assessment, but external, multicenter validation and test-retest reliability data are limited, and availability across languages is uncertain. Similarly, valve-specific “hybrid” add-on item sets have primarily been studied in single-center or pilot settings; large multicenter trials embedding such modules systematically have not yet been reported. Finally, psychosocial issues unique to mechanical-valve recipients (e.g., persistent valve-sound awareness and anticoagulation-related treatment burden) remain incompletely captured by existing disease-specific tools, underscoring the need for validated valve-focused subscales and clearer VHD-specific MCIDs for decision support [26-29].

Comparative Analysis: Psychometric Properties and Domains

The selection of an appropriate HRQoL instrument for patients with VHD depends largely on its psychometric properties, which determine its ability to reliably and accurately capture the patient’s experience in different clinical contexts. Three key psychometric dimensions - validity, reliability, and sensitivity to change - are particularly important when evaluating these tools [30]. Where available, this appraisal cites validations performed in VHD or valve-intervention cohorts; otherwise, evidence is extrapolated from broader cardiac populations, and this is explicitly noted for each instrument.

Validity refers to the extent to which an instrument measures what it is intended to measure. The SF-36 demonstrates strong construct validity in cardiac populations, as it can distinguish between varying degrees of disease severity and correlates well with objective clinical parameters such as New York Heart Association (NYHA) functional class and left ventricular ejection fraction (LVEF) [31]. It also shows good criterion validity when compared with disease-specific instruments [32,33]. The EQ-5D, while validated in numerous cardiac cohorts, has been reported to display ceiling effects in mildly symptomatic patients, limiting its discriminatory capacity [34]. In VHD specifically, SF-36 and EQ-5D have been applied and supported by construct/criterion signals in SAVR/TAVI samples, but many validations originate from mixed cardiac cohorts rather than VHD-only designs; WHOQOL-BREF has limited VHD-specific validation, which partly explains its less frequent use. The WHOQOL-BREF offers broader contextual insight by incorporating environmental and social dimensions, but it may not capture disease-specific limitations in VHD [35]. Disease-specific tools such as the MLHFQ and KCCQ exhibit high content and criterion validity in heart failure populations, and their domains overlap substantially with the symptom profile of VHD [36]. Both MLHFQ and KCCQ have also shown construct/known-groups validity in valve cohorts (e.g., TAVI/SAVR and mitral repair studies), yet formal VHD-only validation programs remain fewer than in heart failure. Ceiling and floor effects are clinically relevant in VHD: high pre-procedure EQ-5D or domain-specific SF-36 scores can obscure meaningful postoperative gains, potentially underestimating benefit and complicating discharge or rehab decisions in lower-symptom or frail patients [23,28].

Reliability assesses the consistency of an instrument’s measurements, whether across items within a single administration (internal consistency) or between different administrations over time (test-retest reliability). SF-36 has excellent internal consistency, with Cronbach’s α values above 0.80 across most subscales in both general and cardiac populations [37,38]. EQ-5D demonstrates high test-retest reliability in stable patients, although its internal consistency is lower because it contains fewer items [39]. MLHFQ and KCCQ consistently report Cronbach’s α values between 0.85 and 0.95, indicating excellent internal consistency, and they maintain strong test-retest reliability, making them suitable for longitudinal follow-up [23]. For WHOQOL-BREF, reliability varies by domain and language version, with cultural adaptation playing a significant role in score stability [24]. For valve-specific and hybrid instruments, internal consistency has been reported in small, single-center series, but test-retest data and multicenter reproducibility are underexplored; large validation efforts and multilingual equivalence studies are still needed. Cross-cultural measurement equivalence also warrants attention: EQ-5D utilities depend on country-specific tariffs, and WHOQOL-BREF domain performance can vary by language/culture, which may affect between-country comparability in multinational valve trials.

Sensitivity to Change (Responsiveness) and MCIDs

KCCQ has demonstrated robust responsiveness after TAVI/TEER and SAVR, and MLHFQ is responsive after valve surgery; however, commonly cited MCIDs (e.g., five points for MLHFQ; five to 10 points for KCCQ) are largely derived from heart failure literature. VHD-specific MCIDs are not yet standardized, so interpreting change should consider procedure type, baseline severity, and frailty. Utility-based measures (EQ-5D, SF-6D) capture broad health changes and support QALY analyses, but in elderly VHD patients with multimorbidity, they may be insensitive to valve-specific improvements (e.g., prosthesis-related issues or nuanced functional gains) and are influenced by non-cardiac comorbidities and the chosen national tariff [3].

**Figure 1:**
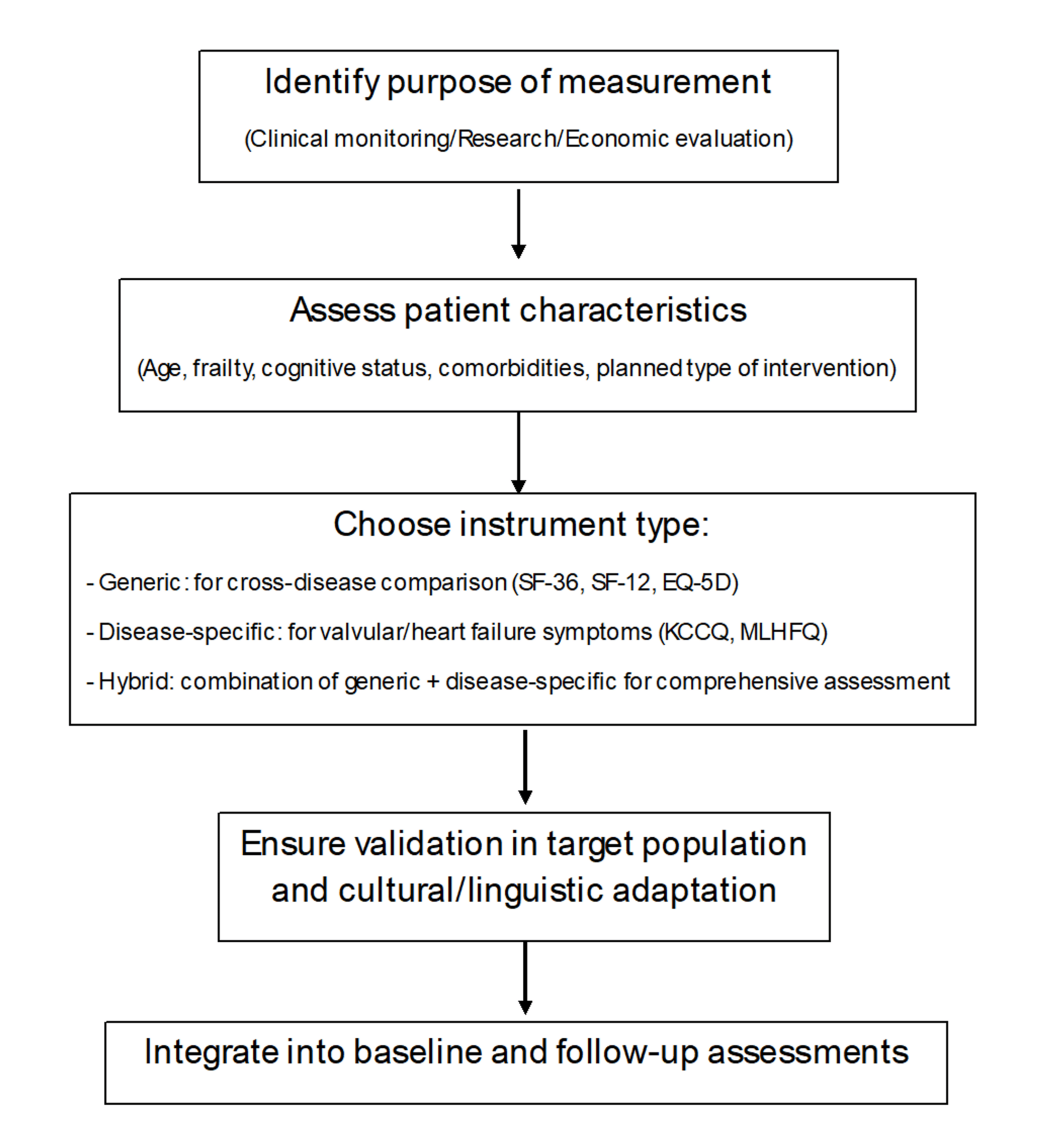
Algorithm of HRQoL Assessment HRQoL: Health-related quality of life.

Overall, the choice of HRQoL instrument in VHD should be guided by the intended purpose of assessment - whether for clinical monitoring, interventional outcome evaluation, or economic analysis - as well as by the balance between comprehensiveness and feasibility in the target patient population. This framework is summarized in Figure 1, while the characteristics of the most widely used instruments are presented in Table 1. For practical implementation, the selection algorithm in Figure 1 is evidence-informed and reflects common use in valve cohorts plus feasibility considerations; it is proposed as guidance rather than a formally validated pathway. In brief: for routine clinical follow-up and shared decision-making, pairing a brief generic utility (e.g., EQ-5D) with a disease-specific tool (e.g., KCCQ-12) is generally preferable; for research requiring granular profiles, SF-36 (± MLHFQ/KCCQ) offers broader domain coverage; and for health-economic evaluations, EQ-5D or SF-6D are appropriate with the above limitations in mind.

**Table 1:** Summary of Generic and Disease-Specific Health-Related Quality of Life (HRQoL) Instruments Used in Valvular Heart Disease SF-36: The Short Form-36 Health Survey; SF-12: The Short Form-12 Health Survey; EQ-5D: EuroQol 5-Dimensions; WHOQOL-BREF: World Health Organization Quality of Life - Brief; MLHFQ: Minnesota Living with Heart Failure Questionnaire; KCCQ: Kansas City Cardiomyopathy Questionnaire. PCS: Physical component summary; MCS: mental component summary; QALY: quality-adjusted life year; VHD: valvular heart disease

Instrument	Type	Domains/Subscales	No. of Items	Score Range	Calculation	Strengths	Limitations
SF-36	Generic	8 domains: Physical Functioning, Role-Physical, Bodily Pain, General Health, Vitality, Social Functioning, Role-Emotional, Mental Health	36	0-100 (per domain and two summary scores: PCS, MCS)	Domain scores transformed to 0-100, higher=better	Widely validated; comprehensive; sensitive to changes; applicable across populations	Lengthy; burdensome for elderly/frail; ceiling/floor effects possible
SF-12	Generic (short form)	Physical and Mental Composite Scores (PCS, MCS)	12	0-100 (PCS and MCS only)	Weighted scoring algorithm	Short, practical; comparable PCS/MCS to SF-36; suitable for surveys/elderly	No individual domain scores; less detail
SF-6D	Generic (short form)	6 dimensions: Physical Functioning, Role Limitation, Social Functioning, Pain, Mental Health, Vitality	6	Utility values 0.29-1.00	Health states (18,000+) valued using population tariffs	Useful in cost-utility and QALY analyses	Less sensitive to small changes; may overweight physical health
EQ-5D	Generic	5 dimensions: Mobility, Self-care, Usual Activities, Pain/Discomfort, Anxiety/Depression + VAS (0–100)	5+1 VAS	Utility index (negative values to 1.00 depending on tariffs)+VAS	Responses mapped to country-specific tariffs+self-rated health scale	Very short; simple; widely used in cost-effectiveness; elderly-friendly	Ceiling effects; limited sensitivity in mild disease; only 5 domains
WHOQOL-BREF	Generic	4 domains: Physical, Psychological, Social Relationships, Environment	26	1-5 per item, transformed to 0-100 per domain	Mean scores of items per domain	Culturally adaptable; broad contextual insight	Less specific to cardiac/VHD; longer than EQ-5D
MLHFQ	Disease-specific (HF/VHD)	Physical, Emotional, Social/Functional impact of HF symptoms	21	0-105 (lower=better)	Sum of item scores (0-5 each)	High internal consistency (α >0.90); responsive after interventions; MCID ≈ 5 points	Not designed for isolated valve disease; focus on HF symptoms
KCCQ (full)	Disease-specific (HF/VHD)	Physical Limitation, Symptom Frequency, Symptom Burden, Self-efficacy, QoL, Social Limitation	23	0-100 (per domain and summary)	Scores normalized to 0-100 (higher = better)	High validity; α >0.80; responsive (MCID 5 points, >10 clinically meaningful); sensitive in TAVI/TEER	More complex scoring; initially HF-specific
KCCQ-12	Disease-specific (short form)	Key domains from KCCQ	12	0-100	Simplified scoring	Shorter, practical for clinics; strong correlation with full KCCQ	Less detail than full version
Valve-specific instruments	Valve-specific	Prosthesis-related anxiety, anticoagulation burden, valve noise, lifestyle impact	Varies (pilot tools)	Varies	Items added to generic tools	Captures unique prosthesis concerns	Early-stage; limited validation; not standardized
Hybrid approaches (e.g., SF-36+valve-specific items)	Mixed	Generic domains + valve-related items	Varies	Varies	Combination scoring	Allows comparability across diseases and valve-specific sensitivity	Non-standardized; preliminary use; needs validation

Contextual Use in Interventions

The selection and application of HRQoL instruments in VHD must be tailored to the type of intervention, the characteristics of the patient population, and the intended purpose of measurement. In surgical valve replacement, which remains the standard treatment for many patients with severe VHD who have low operative risk and good long-term prognosis, HRQoL assessment is often used to evaluate recovery trajectories and compare outcomes between surgical techniques and prosthesis types.

Following surgical valve replacement, substantial improvements in HRQoL are consistently observed, particularly in the domains of physical functioning, reduction in role limitations due to physical health, and decreased bodily pain. These benefits are most pronounced during the first three to six months postoperatively [23]. Improvements in mental health tend to occur more gradually, likely reflecting the psychological adjustment to surgery and adaptation to living with a prosthetic valve [36]. Patients receiving mechanical prostheses may report lower scores in emotional and social functioning due to concerns about anticoagulation, bleeding risks, and lifestyle restrictions, whereas those with bioprosthetic valves often score higher in these domains, benefiting from the absence of lifelong anticoagulation, despite the risk of future reintervention [40]. Where differences between mechanical and bioprosthetic valves are noted, they derive largely from observational cohorts using validated instruments (e.g., SF-36/KCCQ) rather than randomized head-to-head comparisons; accordingly, conclusions should be interpreted as associative rather than causal. Beyond the first postoperative year, HRQoL after SAVR generally stabilizes; domain gains may attenuate in very elderly patients due to competing comorbidities, whereas younger/low-risk surgical cohorts often maintain improvements through 24 months [14,15].

In the case of transcatheter interventions, such as TAVI or TEER, HRQoL evaluation has been critical in demonstrating the patient-centered benefits of these less invasive procedures [41]. TAVI has been extensively studied in randomized trials and real-world cohorts, consistently showing faster and more substantial improvements in HRQoL than surgical aortic valve replacement in the early months after the procedure [14,17]. Gains are most evident in physical functioning, mobility, and self-care, and in some cases these improvements exceed those seen in surgical patients during the initial recovery phase [23]. HRQoL instruments most often used in TAVI studies include the EQ-5D and SF-36 for overall utility and functional domains, as well as KCCQ, which offers disease-specific sensitivity validated in heart failure and valve populations [29,40]. Head-to-head instrument performance suggests that KCCQ is typically the most responsive to early post-TAVI/TEER change, SF-36 provides granular domain profiles but imposes higher respondent burden, and EQ-5D is efficient for utilities/QALYs yet can encounter ceiling effects in lower-symptom patients. Available anchor-based analyses indicate that commonly cited KCCQ minimal clinically important differences (five points small, ~10 points moderate) appear broadly applicable in TAVI/TEER cohorts, but formal VHD-specific MCIDs are not universally established and should be interpreted within procedural context and baseline severity. Longitudinally, many TAVI cohorts maintain net HRQoL gains at 12-24 months; attenuation is more likely in the presence of rehospitalization, advanced frailty, or non-cardiac morbidity [14].

MitraClip therapy for mitral regurgitation, particularly in patients with advanced heart failure or those unsuitable for surgery, has also shown significant HRQoL benefits. The COAPT trial and subsequent observational studies have reported marked improvements in symptom burden, NYHA functional class, and KCCQ scores - often exceeding the threshold for clinically meaningful change - alongside reductions in depression, fatigue, and emotional distress [25,31,42]. Predictors of suboptimal improvement after TEER and TAVI include baseline frailty, cognitive impairment, chronic lung or renal disease, residual valve lesion (e.g., MR/TR or transvalvular gradient), peri-procedural stroke, early readmission, and depressive symptoms; identifying these factors at baseline can inform expectations and rehabilitation planning.

Despite these benefits, not all patients achieve the anticipated improvement in HRQoL after transcatheter therapy. Factors such as pre-existing frailty, severe cognitive impairment, post-procedural complications, or early readmission can limit the gains. Therefore, baseline HRQoL assessment is not only important for measuring treatment effect but also for identifying individuals at risk of suboptimal recovery [43]. In frail or cognitively impaired patients, mode of administration (self vs interviewer-assisted) and non-response bias can affect scores; when direct self-report is not feasible, proxy reporting may be used but should be clearly flagged, analyzed separately, and interpreted cautiously given known proxy-patient discrepancies.

For tricuspid and pulmonary valve interventions, which historically have been less common, HRQoL evaluation is becoming increasingly important as new transcatheter techniques emerge [44]. Severe tricuspid regurgitation is associated with profound functional limitation, and early results from trials such as TRILUMINATE show that transcatheter tricuspid valve repair can produce substantial and sustained improvements in KCCQ scores, as well as gains in physical functioning and emotional well-being [45-47]. In pulmonary valve disease, particularly among adolescents and young adults with congenital heart defects, percutaneous pulmonary valve implantation (PPVI) has been linked to better exercise tolerance, improved emotional role functioning, and higher vitality scores on the SF-36, with benefits persisting at one year [48]. However, for both tricuspid and pulmonary interventions, formal validation of instrument responsiveness and MCIDs within these specific valve populations is still limited; current evidence comes from early trials/series, and larger multicenter validation (including test-retest and cross-cultural performance) remains an unmet need.

Ultimately, the contextual use of HRQoL instruments in VHD interventions requires a balance between comprehensiveness and feasibility. In younger or highly functional patients, more detailed instruments such as the SF-36 may capture subtle changes, whereas in elderly, frail, or cognitively impaired populations, shorter tools like the EQ-5D or KCCQ-12 may be more practical. Combining a generic and a disease-specific instrument often yields the most comprehensive assessment, allowing both cross-population comparison and targeted evaluation of valve-related outcomes [49]. To minimize burden in trials, a pragmatic core set can pair a brief generic utility (e.g., EQ-5D-5L) with a short disease-specific measure (e.g., KCCQ-12), with optional SF-36 administered to a predefined subcohort for detailed domain profiling; assessments at baseline, 30 days, six and 12 months capture early and sustained changes while containing respondent fatigue [14-17].

Challenges and limitations

Despite the growing recognition of HRQoL as a critical outcome in clinical research and patient care, several methodological and conceptual challenges remain, particularly in the context of VHD. One of the most fundamental issues is the subjective nature of HRQoL assessment. Quality of life is inherently personal and shaped by a wide range of factors, including personality traits, cultural background, coping strategies, and socioeconomic status [26]. As a result, two patients with identical clinical characteristics may report very different HRQoL scores, which complicates comparisons both across individuals and between study populations [50]. Age, gender, and educational level further influence how patients interpret and respond to questionnaire items, introducing potential bias unless demographic factors are properly accounted for in analysis [26]. In existing VHD literature, these factors are most often addressed via multivariable linear or mixed-effects models that adjust for age, sex, education, and clinical covariates (e.g., NYHA class, LVEF, frailty); propensity weighting/matching is occasionally used in comparative cohorts. Interaction terms (e.g., age×intervention) are rarely prespecified, and residual confounding remains likely, so covariate-adjusted effect estimates should be interpreted cautiously and, where possible, complemented by stratified summaries.

Many HRQoL tools, particularly generic ones such as the SF-36 and EQ-5D, also suffer from ceiling and floor effects. These occur when patients score at the upper or lower limits of a scale, limiting the ability to detect further improvement or deterioration [30]. This is especially problematic in mild or advanced disease stages, where changes may be clinically meaningful but remain invisible in the score [26]. In VHD cohorts specifically, ceiling effects are more pronounced with generic utilities (notably EQ-5D-3L and, to a lesser extent, EQ-5D-5L) in lower-symptom states, whereas disease-specific tools (e.g., KCCQ, MLHFQ) tend to show fewer ceilings but may exhibit floor effects in very advanced disease. Clinically, this can under- or overestimate response to intervention and affect responder analyses; selecting instruments with headroom at the expected severity level and triangulating with a disease-specific measure mitigates misclassification.

Temporal instability and recall bias present additional challenges. HRQoL scores may vary depending on the timing of assessment, with early postoperative improvements sometimes overestimated due to transient symptom relief, while late complications or psychological adaptation may not be captured if follow-up is short [28]. Retrospective evaluations are also prone to recall errors, particularly in elderly populations [23]. For interventional VHD studies, pragmatic time points that capture trajectory include baseline (pre-procedure), early recovery (discharge or ~30 days), consolidation (three to six months), and maintenance (12 months), with annual assessments thereafter for durability. These intervals balance signal detection (early gains) with late clinical events and remodeling.

Cross-cultural adaptation adds another layer of complexity. While most widely used HRQoL tools have been translated into multiple languages, cultural differences in health perception and communication styles can alter how items are understood and answered. Even within the same country, regional variations in health beliefs may affect responses, emphasizing the need for rigorous cross-cultural validation [44]. For tools relying on country-specific value sets (e.g., EQ-5D), tariff selection materially influences utility estimates and QALY calculations; multinational trials should prespecify tariff strategy and perform sensitivity analyses. WHOQOL-BREF and SF-36 have multilingual versions, but equivalence should be confirmed with formal forward-back translation and measurement-invariance testing in the target VHD population. Illustratively, valve cohorts using Scandinavian KCCQ translations and the HeartQoL instrument after valve surgery report acceptable reliability, yet cross-cultural comparability still requires dedicated testing in diverse settings.

In addition, the lack of consensus on which HRQoL instrument should be standard in VHD research leads to inconsistencies that hinder meta-analyses and direct comparisons between studies [34]. Many studies also rely on single-point measurements, missing the dynamic evolution of HRQoL over time [20]. Finally, the absence of universally accepted minimal clinically important differences (MCIDs) for many tools makes it difficult to judge whether observed changes are meaningful to patients. MCIDs commonly borrowed from heart-failure cohorts (e.g., KCCQ ≈ five to 10 points) are useful heuristics but may not fully reflect VHD-specific trajectories; studies should prespecify anchor-based approaches (e.g., NYHA change, global transition) and include distribution-based sensitivity thresholds to reduce misclassification. Although HRQoL is frequently measured in research, its integration into everyday clinical practice remains limited, partly due to time constraints, lack of training, and insufficient infrastructure for routine administration and interpretation [51]. Additional barriers include variable reimbursement for PROM collection, licensing/translation costs, incomplete EHR integration, device/digital literacy constraints in older adults, and limited clinical feedback loops (e.g., lack of thresholds embedded in workflows). When self-report is infeasible in frail or cognitively impaired patients, proxy reporting can be used but should be clearly flagged and analyzed separately because proxies often rate lower HRQoL than patients [33].

Future directions

Integrating HRQoL measurement into the routine evaluation of VHD patients can enhance patient-centered care and improve clinical decision-making. HRQoL assessment has been recommended both before and after interventions, whether surgical or transcatheter, to track recovery trajectories and identify unmet needs [52]. For busy clinical settings, a feasible bundle is a brief generic utility plus a short disease-specific tool (e.g., EQ-5D-5L+KCCQ-12) at baseline, ~30 days, six and 12 months; this combination balances brevity (≤three to four minutes) with sensitivity to clinically meaningful change. In research trials, a more granular bundle (e.g., SF-36+full KCCQ±EQ-5D/SF-6D for utilities) supports domain-level analyses and cost-effectiveness. The combined use of generic and disease-specific tools can provide a more comprehensive view; for example, pairing the SF-36 with the MLHFQ or KCCQ allows assessment of both global health status and cardiac symptom burden [19,20]. Generic tools alone are rarely sufficient in VHD because ceiling effects may obscure important changes; pairing with a disease-specific measure is recommended whenever possible.

Incorporating HRQoL findings into multidisciplinary heart team discussions ensures that treatment decisions account for the patient’s perspective, especially in elderly or high-risk individuals in whom quality of life may be prioritized over survival benefit [13,35]. Operationalization can follow pragmatic thresholds to prompt discussion (not to replace clinical criteria): for KCCQ, a ≥5-point change is commonly interpreted as minimally important and ≥10-point as moderate; for EQ-5D index, changes of ~0.05-0.10 are often considered meaningful. Discordant patterns (e.g., objective improvement but persistently low HRQoL) should trigger review of comorbidity, frailty, or psychosocial barriers and referral to rehabilitation, rather than alter valve-specific indications per se. Clinician training and the inclusion of HRQoL interpretation in clinical guidelines would further facilitate this integration, enabling providers to use these scores meaningfully in routine care.

From a research perspective, studies should prioritize the evaluation of long-term HRQoL outcomes, as short-term improvements may not reflect sustained benefit [53]. The development of core outcome sets including standardized HRQoL measures for VHD trials has been proposed to improve comparability and strengthen meta-analyses [54]. International efforts (e.g., International Consortium for Health Outcomes Measurement's (ICHOM) heart valve dataset) already outline candidate HRQoL elements and time points; broader adoption and periodic updating will facilitate harmonization across surgical and transcatheter studies. Validation studies tailored to specific VHD subgroups - such as tricuspid or pulmonary valve disease and elderly populations - are also needed [46-48]. Where VHD-specific MCIDs are lacking, trials should pre-specify anchor-based thresholds with sensitivity analyses, rather than importing values wholesale from heart failure cohorts.

Emerging technologies, including digital platforms and mobile health applications, offer opportunities for real-time patient-reported outcome monitoring, enabling earlier detection of deterioration in health status and timely intervention [55]. Practical barriers in elderly/frail populations include device access, digital literacy, visual/cognitive limitations, and caregiver reliance; mitigations include telephone- or paper-fallback options, assisted data entry, larger-font interfaces, and caregiver-proxy modules clearly flagged for analysis. Integration with EHRs (eConsent, automated reminders, score dashboards) improves uptake and data completeness. Combining HRQoL with clinical, imaging, and biomarker data has also been suggested as a strategy to enhance risk stratification and predictive modeling in cardiovascular populations. Standardization should include (i) prespecified time anchors (baseline, 30 days, six and 12 months), (ii) a minimal core of covariates (NYHA class, six-minute walk, LVEF/gradients, NT-proBNP/frailty indices), and (iii) modeling plans (mixed-effects or joint models) that handle missingness and avoid overfitting when HRQoL is combined with imaging/biomarkers.

Finally, several authors have argued that HRQoL should be elevated to a co-primary endpoint in selected patient groups, particularly in frail elderly individuals undergoing TAVI, where survival benefit may be limited [13]. Methodological implications include multiplicity control (hierarchical testing), adequate powering for both endpoints, prespecified MCIDs and responder definitions, and PRO-specific quality safeguards (blinded adjudication of clinical endpoints, rigorous handling of missing PRO data, and adherence to CONSORT-PRO reporting). Regulatory acceptance generally favors HRQoL as a key secondary or co-primary endpoint when justified by patient population and clinical context. Expanding HRQoL assessments to include caregiver outcomes is pertinent but evidence in VHD is sparse; until VHD-specific tools are validated, generic caregiver measures (e.g., Caregiver Strain Index or Zarit Burden Interview) can be considered for exploratory use, with cultural/linguistic validation and clear analytical separation from patient self-report. Expanding HRQoL assessments to include caregiver burden and family impact has also been recommended to capture the broader effects of VHD and its treatment [56].

Limitations of This Narrative Review

As a narrative (non-systematic) overview, several methodological constraints apply. PRISMA methods were not followed, no protocol was pre-registered, and no formal risk-of-bias appraisal or meta-analysis was performed. Database coverage was limited to Medline (PubMed) and Google Scholar. Evidence was prioritized from 2015 to 2025 (with a few foundational older sources), which may introduce recency and citation bias and underrepresent earlier but relevant work. Only English-language publications were considered, creating potential language bias. Comparisons across instruments and interventions are qualitative. Heterogeneity in study designs, follow-up time points, and patient mix (age, frailty, comorbidities) limits direct comparability. Psychometric properties (validity, reliability, responsiveness) are reported as published in the source studies; no re-estimation across cohorts was undertaken. The scope emphasizes aortic and mitral interventions where the literature is largest; evidence for TMVR and contemporary tricuspid therapies is still emerging and likely to evolve rapidly. Pediatric populations were outside the scope (aside from the limited PPVI context), so generalizability to congenital or younger cohorts is restricted.

Despite these limitations, the synthesis is intended to provide a concise, practical orientation for selecting and interpreting HRQoL instruments in VHD.

## Conclusions

HRQoL is now a core outcome in VHD, complementing survival and readmission by capturing how patients feel and function. Across surgical (SAVR) and transcatheter (TAVI, TEER) pathways, HRQoL typically improves early and often remains better at six to 12 months; in many cohorts, magnitude of improvement - especially on disease-specific tools such as KCCQ - tracks with subsequent functional recovery and rehospitalization risk. Nonetheless, discrepancies persist: frailty, multimorbidity, cognitive impairment, and post-procedural complications can blunt HRQoL gains despite acceptable technical results, underscoring the need to interpret scores alongside clinical context.

Instrument choice remains pivotal. Generic measures (EQ-5D, SF-12/36) enable cross-study comparability and health-economic modeling, while disease-specific tools (KCCQ, MLHFQ) provide greater responsiveness around interventions. Important validation gaps remain for VHD-specific use: WHOQOL-BREF has limited valve-focused psychometrics; SF-6D utilities are seldom validated head-to-head against EQ-5D in VHD; MLHFQ and KCCQ are increasingly used in valve cohorts but were not originally designed for valve-specific issues (e.g., prosthesis noise, anticoagulation burden); emerging valve-specific or hybrid instruments (e.g., IDCV) are early-phase with small samples and scarce test-retest data. In addition, HRQoL evidence for emerging therapies such as TMVR and contemporary tricuspid interventions remains nascent and pediatric/PPVI data are not directly generalizable to adults.

HRQoL already influences policy: generic utilities (e.g., EQ-5D) underpin quality-adjusted life-year (QALY) analyses used in coverage and reimbursement deliberations for valve interventions, and international initiatives (e.g., ICHOM heart-valve datasets) are moving the field toward more consistent measurement and time points. Implementation in elderly/frail populations must be pragmatic: digital platforms can streamline collection but face barriers (device access, digital literacy, sensory/cognitive limitations). Hybrid models - brief in-clinic or phone capture with caregiver-assisted or proxy reporting clearly flagged - are often the most feasible.

Going forward, HRQoL should be embedded directly into rehabilitation and follow-up pathways. Practical triggers (e.g., KCCQ declines ≥5-10 points or EQ-5D drops ~0.05-0.10) can prompt targeted actions: referral to cardiac rehabilitation; optimization of diuretics/afterload; screening and treatment for anxiety/depression; anticoagulation counseling; or evaluation for untreated frailty and deconditioning. Caregiver-reported outcomes deserve a defined place in future core sets to capture the broader impact of VHD and its treatments.

The priority directions are as follows.

Standardize measurement: adopt a core bundle (e.g., EQ-5D-5L + KCCQ-12) at baseline, ~30 days, six and 12 months, with clear responder definitions and handling of proxy responses.

Close validation gaps: conduct multicenter psychometric studies in VHD (including TMVR/tricuspid) to establish VHD-specific responsiveness, MCIDs, and test-retest reliability-especially for WHOQOL-BREF, SF-6D, and valve-specific/hybrid tools.

Advance cultural equivalence: ensure language validation and tariff selection are pre-specified for multinational studies; report potential tariff-driven differences in utilities.

Integrate with prognostics: pair HRQoL trajectories with clinical, imaging, and biomarker data in prespecified models to identify poor responders early and personalize rehabilitation.

Enable real-world uptake: deploy low-burden digital/paper workflows within EHRs, with accommodations for frailty and cognitive impairment, and align with guideline/coverage expectations.

By coupling standardized, feasible measurement with rigorous validation and actionable thresholds, HRQoL can move from an adjunct endpoint to a practical lever for decision-making, rehabilitation planning, and policy in the comprehensive care of patients with VHD.
